# Computational study on the polymerization reaction of d-aminopeptidase for the synthesis of d-peptides†

**DOI:** 10.1039/d0ra01138j

**Published:** 2020-05-06

**Authors:** Joan Gimenez-Dejoz, Kousuke Tsuchiya, Ayaka Tateishi, Yoko Motoda, Takanori Kigawa, Yasuhisa Asano, Keiji Numata

**Affiliations:** Biomacromolecules Research Team, RIKEN Center for Sustainable Resource Science 2-1 Hirosawa Wako-shi Saitama 351-0198 Japan keiji.numata@riken.jp; Laboratory for Cellular Structural Biology, RIKEN Center for Biosystems Dynamics Research 1-7-22 Suehiro-cho, Tsurumi Yokohama 230-0045 Japan; Biotechnology Research Center, Department of Biotechnology, Toyama Prefectural University 5180 Kurokawa Imizu Toyama 939-0398 Japan

## Abstract

Almost all natural proteins are composed exclusively of l-amino acids, and this chirality influences their properties, functions, and selectivity. Proteases can recognize proteins composed of l-amino acids but display lower selectivity for their stereoisomers, d-amino acids. Taking this as an advantage, d-amino acids can be used to develop polypeptides or biobased materials with higher biostability. Chemoenzymatic peptide synthesis is a technique that uses proteases as biocatalysts to synthesize polypeptides, and d-stereospecific proteases can be used to synthesize polypeptides incorporating d-amino acids. However, engineered proteases with modified catalytic activities are required to allow the incorporation of d-amino acids with increased efficiency. To understand the stereospecificity presented by proteases and their involvement in polymerization reactions, we studied d-aminopeptidase. This enzyme displays the ability to efficiently synthesize poly d-alanine-based peptides under mild conditions. To elucidate the mechanisms involved in the unique specificity of d-aminopeptidase, we performed quantum mechanics/molecular mechanics simulations of its polymerization reaction and determined the energy barriers presented by the chiral substrates. The enzyme faces higher activation barriers for the acylation and aminolysis reactions with the l-stereoisomer than with the d-substrate (10.7 and 17.7 kcal mol^−1^ higher, respectively). The simulation results suggest that changes in the interaction of the substrate with Asn155 influence the stereospecificity of the polymerization reaction.

## Abstract 2

We studied the molecular mechanism of d-aminopeptidase for the synthesis of polypeptides incorporating d-amino acids.

## Graphical abstract

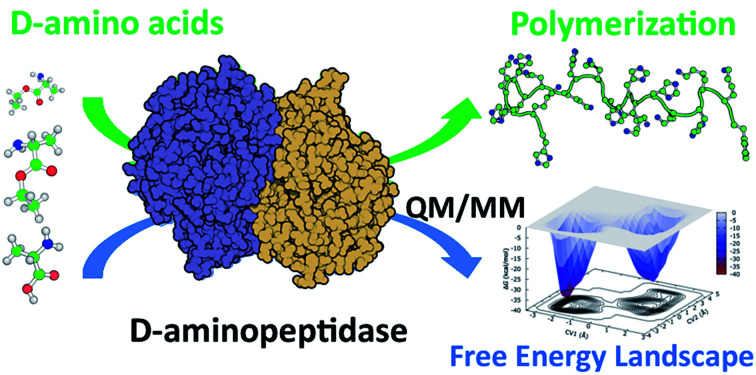

## Introduction

Of the 20 canonical proteinogenic amino acids, all except Gly have a chiral centre at the Cα backbone atom, resulting in the presence of l- and d-stereoisomers. Every living organism exclusively uses l-amino acids for constructing proteins. Although the biological occurrence of d-amino acids is rare, their presence has been reported in bacterial cell walls as components of peptidoglycan and other periplasmic extracellular polymers,^1^ in antibacterial and antifungal peptides,^2,3^ in the cellular fluids of some invertebrate marine worms and shellfish,^4^ in the venom of some spiders^5^ and platypuses^6,7^ and in the skin secretions of some amphibians acting as homologs of mammal neurotransmitters and hormones.^4,8^ The discovery of these peptides and their compelling biological functions has drawn increasing interest to the incorporation of d-amino acids into polypeptides.

The interest in polypeptides and peptides in various fields is increasing due to their diverse physical properties, biological functions and promising applications, such as in bio-based materials and as green alternatives to petroleum-derived plastics, pharmaceutical agents, therapeutic drugs, drug delivery agents, and antimicrobial peptides.^9–11^ In particular, interest in peptides as delivery agents and inhibitors has arisen because they are highly specific for their targets and have high biocompatibility and low toxicity.^10,12^ However, peptides have low biostability because they are prone to hydrolysis by proteases, reducing their half-life in circulating plasma, and they can be immunogenic, which severely limits their application as therapeutic agents *in vivo*.^12,13^

Using peptides with d-amino acids in their structure is a highly promising strategy for overcoming these limitations. d-Amino acids can alter the secondary structure relative to that generate with all l-amino acids, which is the structure widely recognized by proteases. They can adopt different backbone *φ* and *ψ* angles that are disfavoured by l-amino acids, stabilizing specific structural conformations that cannot be assumed by l-amino acids. The different configurations available with d-amino acids can confer favourable biological and chemical proprieties to peptides and proteins. d-Amino acids can stabilize α-helices,^14^ β-turns and β-hairpins^15,16^ and promote novel peptide topologies. The α-helices in proteins made entirely of l-amino acids are nearly always right-handed. On the other hand, it has been observed that proteins formed exclusively from d-amino acids are the mirror image of their all l-amino acid counterpart.^17,18^ Thus, artificial proteins made of d-amino acids form left-handed α-helices, changing the conformation of the secondary structure.^19^ Therefore, d-peptides are highly resistant to proteolysis, increasing their biological half-life and biostability^20–23^ and resulting in low immunogenicity, as they are not recognized by other proteins.^24^ Furthermore, short d-peptides can be administered orally, and they are systemically absorbed; in contrast, l-peptides have to be injected to avoid digestion.^25^ For these reasons, the incorporation of d-amino acids into peptides and proteins has many potentially benefits.

**Scheme 1:**
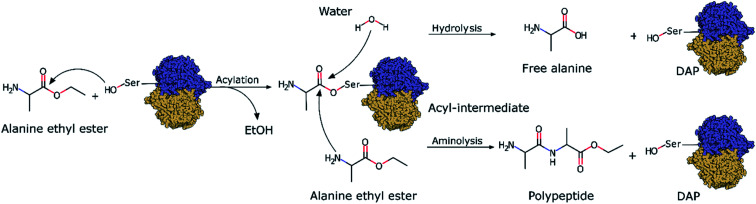
DAP-mediated polymerization showing the acylation, aminolysis and hydrolysis reactions.

Although the incorporation of d-amino acids into polypeptides presents several advantages, neither chemical synthesis nor chemoenzymatic peptide synthesis have been efficient enough for their synthesis or incorporation into peptides. Chemical synthesis strategies face problems regarding the size of the peptides they can produce, as these strategies require multiple difficult and complex protection and deprotection steps^26^ as well as toxic chemicals and solvents.^27^ On the other hand, chemoenzymatic peptide synthesis is based on the use of protease enzymes as biocatalysts to synthesize polypeptides *via* the aminolysis of amino acids (Scheme 1). Under physiological conditions, proteases do not catalyze polymerizations since the thermodynamic equilibrium of the reactions strongly favours hydrolysis. Thus, in chemoenzymatic synthesis, polymerization is strongly dependent on fundamental reaction parameters, especially substrate concentration.^28^ This technique affords high yields, is atom economical, can be performed under mild conditions, and does not require organic solvents.^29^ However, one major drawback is that the synthesis is restricted by the natural substrate specificity of proteases and they generally only recognize l-amino acids. Thus, the use of proteases as biocatalysts is limited by their overall lack of d-stereospecificity and inability to recognize d-amino acids. As recently explored in our previous work with quantum mechanics/molecular mechanics (QM/MM) simulations,^30^ the stereospecificity showed by papain, an enzyme that only polymerizes l-amino acids, was mainly influenced by the chiral conformation adopted by the acyl intermediate with the substrate (l- or d-Ala-OEt). The simulations revealed that the aminolysis of l-Ala-OEt by papain had lower energetic barriers than that of the reaction of d-Ala-OEt,^30^ its energetic barriers for aminolysis and hydrolysis are comparable, and therefore, the reactions are competitive. However, the aminolysis reaction with d-acyl intermediates has higher energetic barriers, and because hydrolysis is much more energetically favourable, the polymerization of the d-amino acids into d-peptide does not occur.

Here, we investigate one of the few known d-stereospecific proteases, d-aminopeptidase (DAP) (EC 3.4.11.19, MEROPS Family S12), to develop chemoenzymatic polymerization reactions of d-amino acids. DAP is a serine protease isolated and purified from *Achromobacter anthropic* by Asano *et al.*, and it has a molecular weight of ≈110 kDa and consists of 2 identical subunits.^31^ Based on the conserved elements in its amino acid sequence (the Ser–X–X–Lys and Tyr–X–Asn conserved motifs, where X can be any amino acid), its primary structure is similar to β-lactamases, R61 d-carboxypeptidase and penicillin-binding proteins, and its activity is inhibited by β-lactam antibiotics, and hence DAP is considered a penicillin-recognizing protein.^32–34^ Indeed, its active site presents high structural homology with the active sites of class A and C β-lactamases and penicillin-binding proteins, and the residues Ser62, Lys65, Tyr153, Asn155, His287 and Gly289 are conserved. Among these residues, Ser62 and Lys65 are essential for catalytic activity as confirmed by site-directed mutagenesis,^31^ whereas Tyr153, located near these residues, stabilizes a hydrogen-bond network.^33,35^ Regarding the peptidase activity, DAP presents remarkable stereospecificity towards amide bonds of d-amino acids, especially low-molecular-weight d-peptides, d-amino acid esters, and peptides with d-alanine at the N terminus.^31^ Due to this specificity for d-amino acids and because it is inhibited by β-lactam antibiotics, this enzyme may have a physiological role in the biosynthesis or degradation of peptidoglycans.^31,36,37^ Additionally, DAP was reported to be able to synthesize d-alanine oligopeptides from d-alanine methyl esters in organic solvents.^38^

Using DAP as a model enzyme of d-stereospecific proteases, we tried to identify the elements important to its stereospecificity. We hope that these results can be used to rationally modify the active sites of enzymes to increase their specificity and usefulness, which will facilitate the synthesis of more biostable peptides through the incorporation of d-amino acids, the creation of new peptide-derived biomaterials,^20,21^ and the synthesis of fully d-peptides for testing as new pharmaceuticals.^13,39^ Additionally, having access to d-stereospecific proteases could increase the number of accessible peptide structures for researchers, reduce their synthesis costs, and make peptides more easily available for exploration and use in new biotechnologies, such as in gene or protein delivery systems targeting cells or organelles,^40,41^ in cell-penetrating peptides or in peptide-based antibiotics.^42–44^

## Experimental

### Materials

l-Alanine ethyl ester hydrochloride (l-Ala-OEt) and d-alanine ethyl ester hydrochloride (d-Ala-OEt) were purchased from Sigma-Aldrich (St. Louis, MO, U.S.A.). l-Proline benzyl ester (Pro-OBzl) was purchased from Tokyo Chemical Industry (Japan). Deuterium oxide was purchased from Cambridge Isotope Laboratories, Inc. Standard chemicals were purchased from Wako Chemical Co. (Kanagawa, Japan).

### Synthesis of d-alanine dipeptide ethyl ester as standard

To a dispersion of d-Ala-OEt (0.768 g, 5 mmol), *N-tert*-butoxycarbonyl-d-alanine (Boc-d-alanine) (0.946 g, 5 mmol), and 1-hydroxybenzotriazole monohydrate (HOBt, 0.766 g, 5 mmol) in chloroform (10 mL) in a flask equipped with an addition funnel and a stopcock was added triethylamine (0.70 mL, 5 mmol) at 0 °C under nitrogen (Fig. S1†). A solution of 1-ethyl-3-(3-dimethylaminopropyl)carbodiimide hydrochloride (0.959 g, 5 mmol) in chloroform (10 mL) was added dropwise to this mixture, and the solution was stirred at −10 °C for 30 minutes and then at 25 °C for 18 h. After the reaction, the solution was washed with 5% sodium hydrogen carbonate aq. solution and brine. The organic layer was dried with magnesium sulfate and concentrated with a rotary evaporator. After drying under vacuum, the Boc-protected dipeptide ester Boc-d-Ala-d-Ala-OEt was obtained as a white solid. Then, the solid was dissolved in dichloromethane (5 mL), and trifluoroacetic acid (1.9 mL, 25 mmol) was added at 0 °C under nitrogen, and the solution was stirred at 25 °C for 6 h. After the reaction, the solvent was removed by vacuum distillation. The crude viscous liquid was mixed with dioxane/HCl (4 M, 2.5 mL) and poured in excess diethyl ether. The white precipitate was separated by filtration, washed with diethyl ether, and dried under vacuum to give d-Ala-d-Ala-OEt as the hydrochloride salt. The yield was 0.67 g (60%).

### d-Aminopeptidase expression and purification

d-Aminopeptidase from *Ochrobactrum anthropi* SCRC C1-38 cDNA was kindly provided by Prof. Asano. DAP was synthesized and purified using a reported previously cell-free protein expression system.^45^ A cell-free dialysis was performed on a large scale (36 mL) using a dialysis membrane with a molecular weight cutoff (MWCO) of 15 kDa (Pierce, Rockford, IL) using the reaction conditions reported previously.^46^ The internal solution was dialyzed in a dialysis tube (Spectra/Por 7, MWCO of 15 kDa, Spectrum) against the external solution at 30 °C for 16 h with shaking.^47^ The internal solution with the tagged protein was purified by AKTA Xpress (GE Healthcare, Little Chalfont, U.K.) using a previously reported procedure.^46^ Briefly, the protein solution was purified on a HisTrap column (5 mL, nickel–nitrilotriacetic acid (Ni–NTA) column, GE Healthcare). Sodium dodecyl sulfate-polyacrylamide gel electrophoresis (SDS-PAGE) was performed using 15 to 20% precast Tris–HCl gels (DRC Co. Ltd, Kyoto, Japan). The gel was stained with Coomassie brilliant blue (Fig. S1c†).

### Chemoenzymatic polymerization assays

The polymerization reactions with l- or d-Ala-OEt were based on previously reported reaction conditions.^48^ The reactions were conducted at 25 °C in a final volume of 1 mL of 1 M PBS buffer (pH 8.0), containing 0.01 mg mL^−1^ DAP and 0.5 M of l- or d-Ala-OEt monomer as the substrate. The reactions were allowed to proceed for 1 minute under shaking. In the control reactions, no enzyme was added to the assay. The reactions were immediately transferred to an ice-cold bath and centrifuged at 10 000*g* for 10 minutes in an Amicon 10K tube at 4 °C to remove the enzyme. The samples were analysed by RP-HPLC.

### RP-HPLC analysis

RP-HPLC system consisted of an auto sampler (AS-2055, JASCO, Tokyo, Japan), a gradient pump (PU2089, JASCO) a column oven (CO-4060, JASCO), and a C18 column (YMC-Triart C18, particle size 5 μm, 150 × 4.6 mm i. d., YMC, Kyoto, Japan). The samples were injected into the HPLC system in the mobile phase, which was composed of 0.1 M PBS buffer (pH 8) as eluent A and acetonitrile as eluent B. The composition of the mobile phase was linearly changed from 98% A and 2% B to 90% A to 10% B over 30 minutes and was used at a flow rate of 1 mL min^−1^. The elution of the various components was monitored by UV absorbance at 220 nm. The peak areas and retention times were quantified with the chromatography software (ChromNAV, JASCO, Tokyo, Japan) and corrected relative to an internal standard (l-proline benzyl ester (H-Pro-Bzl)). The concentration of the product was calculated by comparison to a calibration curve that was prepared by plotting the peak areas of the standard d-alanine dipeptide ethyl ester against the concentration.

### Nuclear magnetic resonance

^1^H NMR spectra were recorded on a Varian NMR 500 (500 MHz) spectrometer (Varian Medical Systems) at 25 °C and acquisition was controlled with the VnmrJ software. Sixty-four scans were taken during each NMR experiment. Data were processed and analyzed with ACD/NMR Processor Academic Edition, version 12.01 (Advanced Chemistry Development, Inc.).

### Model preparation and classical molecular dynamics simulations

The crystallographic structure of DAP (PDB ID 1EI5)^33^ was used for the simulations. The substrates l-Ala-OEt and d-Ala-OEt were first docked into DAP with the docking program AutoDock Vina version 1.1.2.^49^ Before docking, all ligands and water molecules were removed from the PDB file. Polar hydrogens were added to ligands and receptor by using the Hydrogen module in AutoDock Tools version 1.5.6,^50^ then Gasteiger united atom partial charges and atom types were assigned and PDBQT files generated. The protein molecule was kept rigid, while all the torsional bonds in the substrates were set free to rotate. A 15 Å docking box around the oxygen atom of Ser62 was defined. The best docking conformations of the substrates were used as initial configuration for the MD. The protonation state of the amino acid residues for the MD was set according to the environment and previous studies; for modelling the acylation reaction, Lys65 was set as neutral, and nearby residue His287 was kept neutral and ε-protonated.^35,51^ All molecular dynamic simulations were performed using AMBER 16.^52^ The atoms in the protein were described using the AMBER ff14SB force field,^53^l- and d-Ala-OEt substrates were described with a GAFF,^54^ and the system was solvated with TIP3P water molecules.^55^ A time step of 2 fs along with the SHAKE algorithm was used,^56^ while the particle-mesh Ewald (PME) method^57^ was used to calculate long-range interactions. The systems were equilibrated by a series of gradual-step simulations in which distance restraints were set between the substrate molecules and the protein to maintain the important interactions. The simulations were extended until the system was considered equilibrated according the root mean square deviation (RMSD) (Fig. S2†). The relaxed structures obtained from the last part of the trajectories were used as the starting structures for hybrid QM/MM calculations. The trajectories were analysed using CPPTRAJ^58^ and VMD software.^59^

### QM/MM molecular dynamics simulations

Hybrid QM/MM molecular dynamics simulations were carried out as previously described.^30^ Briefly, we used density functional theory (DFT) B3LYP/6-31G* basis set^60^ to describe the atoms in the QM system. The QM calculations were carried out using Gaussian09 (ref. 61) together with the AMBER/Gaussian interface.^62^ The integration time step for the QM calculations was 0.2 fs. The explicit link atom approach, implemented in sander, the molecular dynamics program of AMBER, was used to separate the QM and the MM regions when their boundary crossed covalent bonds.^63,64^ The electrostatic interactions between the MM and QM regions were truncated at a cutoff of 8 Å.^62^

### QM/MM adaptively biased molecular dynamics

The free-energy landscapes (FELs) of the acylation and aminolysis reactions were determined using adaptively biased molecular dynamics (ABMD).^65,66^ This enhanced sampling technique based on metadynamics^67,68^ introduces a time-dependent biasing potential in the simulation, enabling the exploration of energy surfaces of selected collective variables (CV). In our study, the flooding timescale of the bias deposition was set at 30 fs (150 MD steps), and the resolution was set at 1 kcal mol^−1^. The first crossing criterion was used to determine the end of the simulation, as recommended for chemical reactions.^69^ Walls at 3.5 Å between substrates/products and Ser62 were used to reduce the FEL space of the chemical event and reduce computational effort.

### QM/MM ABMD simulations of the acylation reaction

**Fig. 1:**
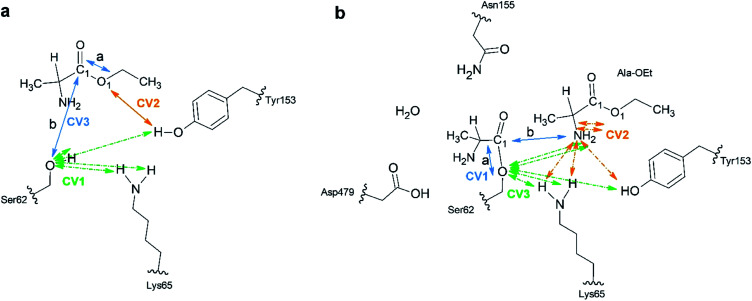
Schematic of the atoms treated at the B3LYP/6-31G* QM level of theory. (a) Acylation reaction; the catalytic residues Ser62, Lys65 and Tyr153 together with the l- or d-Ala-OEt substrates were subjected to QM modelling. Green arrows indicate the atoms used in multiple bonds interaction in CV_1_. The orange arrows indicate CV_2_ and the blue arrows show the distances *a* and *b* in used as LCOD in CV_3_. (b) Aminolysis reaction; all the represented atoms were subjected to QM modelling. CV_1_ LCOD are indicated by blue arrows, the interactions present in CV_2_ are shown with orange arrows and the atoms involved in interactions in CV_3_ are indicated with green arrows. Link atoms are marked with curved lines.

A total of 64 atoms (including 3 H link atoms) were treated quantum mechanically in the acylation reaction with the putative systems while 86 300 atoms were treated with MM. Three CVs (CV_1_, CV_2_ and CV_3_) were used to describe the acylation reaction. CV_1_ is the number of bonds between the O atom of Ser62 and the H atoms of O_Tyr153_ and N_Lys65_ (Fig. 1a). CV_2_ was defined as the distance between the O1_Ala-OEt_–H_Tyr153_ atoms. CV_3_ is defined as a linear combination of distances (LCOD) in the form of the distance between bond breaking minus bond forming in the reaction: the distance between the C1_Ala-OEt_–O1_Ala-OEt_ atoms (a) minus the distance between the C1_Ala-OEt_–O_Ser62_ atoms (b) (CV_1_ = *d*(a) − *d*(b)). CV_1_ was selected because it samples the proton transfer between Ser62 to any nearby base. CV_2_ was chosen to model the proton transfer from Tyr153 to O1 of Ala-OEt to form ethanol. CV_3_ was selected to accelerate the nucleophilic attack of the Ser62 O to Ala-OEt and the breaking of the ester bond between the C1 and O1 atoms of Ala-OEt (Fig. 1a).

### QM/MM ABMD simulations of the aminolysis reaction

For the aminolysis reaction, a total of 94 atoms (including 5 H link atoms and a water molecule) were treated at the QM level of theory (B3LYP/6-31G* basis set) while 86 411 atoms with MM. The following CVs were used to model the reaction. CV_1_ was the LCOD between the C1_Acyl-interm._ and O_Ser62_ atoms (a) minus the distance between the C1_Acyl-interm_ and N_Ala-OEt_ atoms (b) (CV_1_ = *d*(a) − *d*(b)) (Fig. 1b). CV_2_ was defined as the number of bonds between the N atom of the attacking Ala-OEt and the H atoms of N_Ala-OEt_, O_Tyr153_ and N_Lys65_. CV_3_ was defined as the number of bonds from O_Ser62_ to the H atoms of N_Ala-OEt_, O_Tyr153_ and N_Lys65_. CV_1_ was chosen to sample the nucleophilic attack of the N atom of Ala-OEt to the C1 atom of the acyl intermediate and its cleavage from the O atom of Ser62. CV_2_ was selected to model the proton transfer from one of the protons of the attacking nucleophile to any nearby group. CV_3_ was selected to model the transfer of a proton to the O atom of Ser62 (Fig. 1b).

## Results and discussion

### DAP-mediated polymerization activity in aqueous buffer

**Fig. 2:**
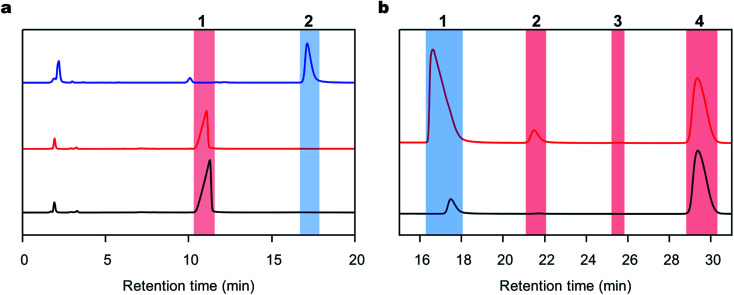
Representative RP-HPLC chromatograms of the polymerization reaction. (a) RP-HPLC chromatograms of the standards used to characterize the reactions. The chromatograms of the l-Ala-OEt, d-Ala-OEt and d-alanine-OEt dipeptide standard are shown in black, red and blue lines, respectively. (1) The peak for l- or d-Ala-OEt (the substrate) at a retention time of 11–12 minutes. (2) The peak of the d-alanine dipeptide product appeared at a retention time of 16–17 minutes. (b) Representative RP-HPLC chromatograms of the reactions performed with DAP with l- or d-Ala-OEt, shown in black and red lines, respectively. (1) The peak of the l- or d-Ala-Ala-OEt products formed in the reaction. (2 and 3) Possible peaks of d-alanine ethyl ester tri- and tetrapeptides, respectively. (4) Peak of the internal standard used in the reaction.

DAP was synthesized using a 36 mL-scale cell-free protein synthesis method and was purified by affinity chromatography, resulting in a final protein yield of 48.3 mg (Fig. S1c†). We polymerized the peptides with DAP under mild conditions, namely, 1 M PBS buffer at pH 8.0 and 25 °C in the absence of any organic solvent based on our previous procedure for other enzymes.^29,48^ Reactions were carried out with l- or d-Ala-OEt, and the soluble products of these reactions were quantified and analyzed by RP-HPLC. To identify the retention time of the product peak and quantify the rate of the reaction, d-alanine dipeptide ethyl ester was chemically synthesized and used as a standard (Fig. S1a†). The reactions were conducted using Boc-d-alanine and d-Ala-OEt in chloroform, and the residue was purified to afford a white powder in 60% yield. The synthesis of d-alanine dipeptide ethyl ester was confirmed by ^1^H NMR (Fig. S1b†). d-Alanine dipeptide ethyl ester, together with l-Ala-OEt and d-Ala-OEt, were used to determine the retention time of each substance in the HPLC chromatograms (Fig. 2a). DAP is able to polymerize l- and d-Ala-OEt under aqueous conditions (1 M PBS, pH 8.0) without a tertiary amine or organic solvent (Fig. 2b).^38^ Similarly, as reported previously for other enzymatic activities,^31,32^ DAP displayed a higher polymerization efficiency for d-substrates (d-Ala-OEt ∼4000 U mg^−1^) than for l-substrates (l-Ala-OEt activity ∼150 U mg^−1^). In addition, additional peaks appeared in the HPLC chromatograms of the reactions with d-Ala-OEt, conceivably corresponding to the trimer and tetramer (Fig. 2b).

### QM/MM acylation reaction

To understand the unique stereospecificity of DAP and confirm its proposed mechanism,^35^ we carried out QM/MM ABMD simulations since this simulation method is a powerful tool for elucidating the stereospecificity of protease enzymes in the polymerization of amino acids.^30^ The equilibrated structures of DAP and the substrates were obtained after performing classical MD simulations. The catalytic triad is formed by the residues Ser62, Lys65 and Tyr153, which are in close proximity. The catalytic Ser62 oxygen forms a hydrogen bond with the amide group of Lys65, which in turn forms a hydrogen bond with Tyr153.^33,35^ Moreover, based on QM studies with closely related enzymes of the same family with similar catalytic residues (penicillin-binding proteins and β-lactamases),^51,70,71^ Lys65 in the catalytic triad was set as neutral, as it is considered to act as the general base for extracting the proton of Ser62. After equilibration, the amino group of the d-Ala-OEt substrate interacts with Asn155 and the oxygen group in the main chain of Ala480, while in the simulation with l-Ala-OEt, this residue interacts with the oxygen of the main chain of Ala288. Additionally, the methyl group of d-Ala-OEt is located in a hydrophobic pocket close to the residues Ala288, Ala480, and Trp220 (Fig. S3†).

The acylation reaction was modeled using three CVs (Fig. 1a) to drive the reaction from the initial reactant state to the acyl intermediate. CV_1_ includes the number of bonds from the Ser62 oxygen to the hydrogens of Ser62, Lys65 and Tyr153, which accelerate proton transfer from Ser62 (Fig. 1a in green). CV_2_ takes into account the transfer of a proton from Tyr153 to the ester group of Ala-OEt to form ethanol (Fig. 1a in orange). CV_3_ samples the nucleophilic attack of the Ser62 O on C1 of the substrate and the cleavage of the ester bond of the substrate (Fig. 1a in blue). Attempts to model the acylation reaction with similar CVs used previously to model the same reaction^30^ were not successful.

**Fig. 3:**
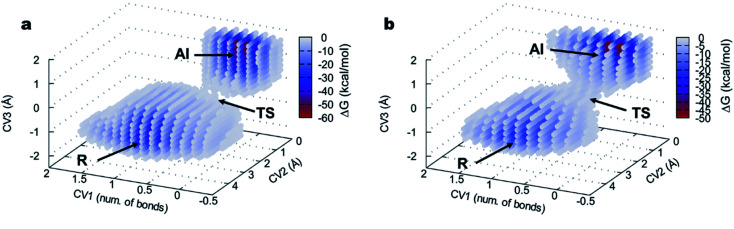
FEL of the DAP-catalyzed acylation reaction visualized as three-dimensional surfaces for (a) l-Ala-OEt and (b) d-Ala-OEt. CV_1_ (number of bonds), CV_2_ and CV_3_ are represented in *x*, *y* and *z* axes, respectively. R, reactants; TS, transition state; AI, acyl intermediate.

The 3D FELs obtained from the QM/MM simulations comparing the acylation reactions with l- and d-Ala-OEt are shown in Fig. 3a and b, respectively. The shapes of the FELs of the reaction with both substrates indicate a concerted mechanism with two energy minima. One is situated at the bottom of the FEL corresponding to the reactants (R), and the other is located at the top of the acyl intermediate (AI), and they are separated by a transition state (TS) (Fig. 3). The acylation reaction with d-Ala-OEt presents an energy barrier of 26.5 kcal mol^−1^ (Table 1) and a R minimum with an energy of −30.2 kcal mol^−1^. On the other hand, the DAP-mediated acylation with l-Ala-OEt presents a free energy barrier of 37.2 kcal mol^−1^, which is 10.7 kcal mol^−1^ higher than that with the d-amino acid (Table 1). The collective variables used to model the acylation reactions l- and d-Ala-OEt changed from CV_1_ ∼1 bonds, CV_2_ of approximately 3 Å and CV_3_ of ∼−1.5 Å in the R, to be placed in the AI at approximately 0.2 bonds for CV_1_, ∼1 Å for CV_2_ and approximately 1.5 Å for CV_3_. These changes take place because in the AI, the Ser62 oxygen has transferred its proton and is bound to C1, the proton of Tyr153 is covalently bound to the O1 atom making ethanol (CV_2_), the distance between the oxygen of Ser62 to C1 is shorter to allow formation of the acyl intermediate (CV_3_a), and the C1–O1 distance of Ala-OEt was increased due to the cleavage of the ester bond (CV_3_b).

**Table 1:** Calculated free energy barriers (kcal mol^−1^) for the acylation and aminolysis reactions, and the energy difference between the barriers for the l- and d-Ala-OEt isomers

DAP	l-Ala-OEt	d-Ala-OEt	Δ*G*l–d
Acylation	37.2	26.5	10.7
Aminolysis	44.5	28.8	17.7

**Fig. 4:**
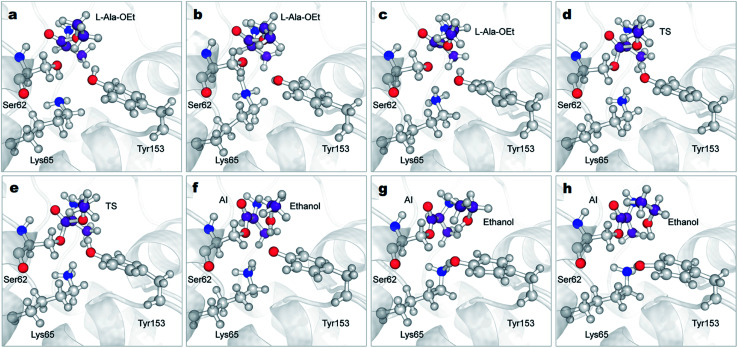
Mechanism of the acylation reaction of DAP and l-Ala-OEt obtained from QM/MM ABMD simulations. Time-course results are shown from (a)–(h). DAP is represented in grey cartoon, atoms treated QM (Ser62, Lys65, Tyr153 and L-Ala-OEt) are shown in ball and stick representation. The carbon atoms of the L-Ala-OEt are shown in purple color. AI, acyl-intermediate; TS, transition state.

The reactions with both substrates follow the same mechanism; they start with the transfer of the proton of Ser62 to neutral Lys65, protonating this residue (Fig. 4a–c, S4 green line and S5a–c†). This proton extraction is followed by the attack of the Ser62 oxygen on C1 atom of Ala-OEt and the shortening of the distance between the Tyr153 proton and the oxygen of the ester of Ala-OEt, bringing them quite close (Fig. 4d, e, S4b and d blue dotted line and S5d and e†). Afterward, the ester bonds of the tetrameric species elongate to the point of cleavage, and the proton of Tyr153 is transferred to the leaving group, releasing the newly formed ethanol molecule (Fig. 4e, f, S5e and f†). Finally, now-protonated Lys65 comes closer to deprotonated Tyr153, and a proton is transferred to negatively charged Tyr153, neutralizing it (Fig. 4g, h and S5†). The acylation mechanism observed in this work is analogous to the mechanism proposed by Khaliullin and collaborators.^35^ One difference observed in the CVs as a function of time (Fig. S4†) is that, in the reaction with d-Ala-OEt, CV_3_b monotonically decreases from 3 Å to ∼1.4 Å. That is, the distance between the C1⋯O1 atoms decreases until a covalent bond is formed (Fig. S4d†), and the distances in CV_2_ are short. CV_1_ (the number of bonds to the Ser62 oxygen) only changes after CV_3_b begins to decrease. With l-Ala-OEt, the CV_3_b distance is already approximately 2 Å and only decreases after the sharp drop of CV_1_ (Fig. S4b†) from ∼1 to 0 bonds, followed also by a sharp drop in CV_2_. This suggests that with d-Ala-OEt, the reaction occurs more cooperatively than with l-Ala-OEt, in which the driving event seems to be the attack of Ser62 to the C1 atom of the substrate. Previous QM studies with class-C β-lactamase enzymes, which have similar active sites, showed that the barriers to the acylation of its inhibitors were 22 kcal mol^−1^ and 17 kcal mol^−1^ for aztreonam and cephalothin,^72^ respectively, and 25 kcal mol^−1^ for avibactam.^71^ The free energy barrier of the acylation of DAP with d-Ala-OEt is similar to those reported for inhibitors. However, l-Ala-OEt presents a higher energy barrier for acylation than d-Ala-OEt and the inhibitors modeled in other studies. The difference in the energy barrier between the l- and d-Ala-OEt substrates could be explained by the interaction between the amino group of d-Ala-OEt and Asn155. On the other hand, l-Ala-OEt does not form an interaction with Asn155, and its amino group is located near the main-chain oxygen of Ala288.

### QM/MM aminolysis reaction

After modeling the acylation reaction, we simulated the aminolysis reaction starting from the acyl intermediate enzyme. We treated the same atoms used to model the acylation, that is, the catalytic triad, at the QM level of theory, and added the formed acyl intermediate and a new Ala-OEt monomer acting as a nucleophile. However, all attempts to model the reaction were unsuccessful or presented unreasonably high energetic barriers (>60 kcal mol^−1^). During the equilibration of the system with classical MD, we observed that the loop on which Asp479 is located moved closer to the acyl intermediate and that Asp479 stabilized the acyl intermediate through a H-bond to its amino group. Asn155 has also been reported to be conserved in the active sites of proteins in the same family as DAP.^34^ Additionally, we observed that a water molecule was located in the catalytic site close to Asp479, Asn155 and the amino groups of the attacking Ala-OEt nucleophiles and the acyl intermediate (Fig. S6†). Thus, we incorporated the Asn155 and Asp479 residues and the water near these residues into the QM system to model the aminolysis reaction.

The only CVs we found that were able to carry out the aminolysis reaction are shown in Fig. 1b. CV_1_ increased the sampling of the nucleophilic attack of Ala-OEt to the C1 atom of the acyl intermediate, and the separation of the acyl intermediate from Ser62 is given by an LCOD (Fig. 1b in blue). CV_2_, as the number of bonds to the N atom of Ala-OEt, is correlated with the acceleration of the transfer of one of the protons of the amino group of Ala-OEt to a nearby acceptor atom (Fig. 1b in orange). CV_3_, as in CV_1_ in the acylation reaction, represents the number of bonds between the Ser62 oxygen and the nearby H atoms, and these bonds accelerate the transfer of a proton to Ser62 to regenerate the enzyme (Fig. 1b in green).

**Fig. 5:**
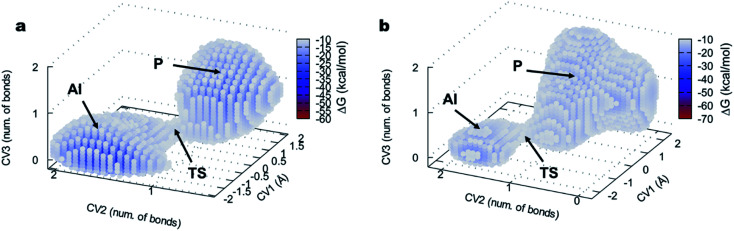
FEL of the DAP-catalyzed aminolysis reaction visualized as three-dimensional surfaces for (a) l-Ala-OEt and (b) d-Ala-OEt. CV_1_ is a LCOD and CV_2_ and CV_3_ are number of bonds, represented on the *x*, *y* and *z* axes, respectively. AI, acyl intermediate; TS, transition state; P, product. For clarity, only points with an energy lower than −10 kcal mol^−1^ are shown.

The free energy landscapes of the aminolysis reactions with l- and d-Ala-OEt as substrates are presented in Fig. 5. The aminolysis with l-Ala-OEt presents an energy barrier of 44.5 kcal mol^−1^, which is higher than the energy barrier with d-Ala-OEt (28.8 kcal mol^−1^, Table 1). Thus, there is a greater difference between the energy barriers of the aminolysis reactions with the different substrates, 17.7 kcal mol^−1^, than there was between the acylation reactions, for which the difference between the barriers with the Ala-OEt isomers was 10.7 kcal mol^−1^. Thus, we concluded that aminolysis is the limiting step of the polymerization reaction for both substrates.

**Fig. 6:**
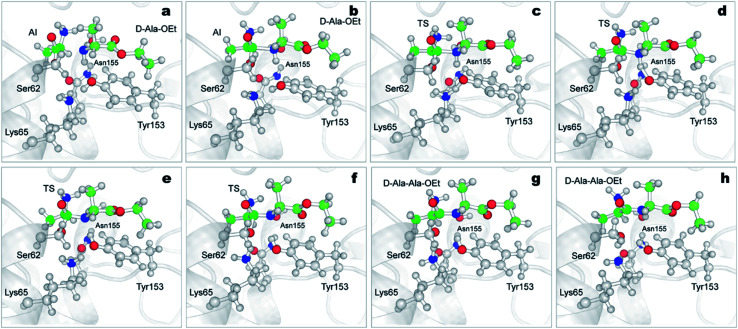
Mechanism of the aminolysis reaction of DAP with Ser62-acylated and d-Ala-OEt obtained from QM/MM ABMD simulations. Time-course results are shown from (a)–(h). DAP is represented in grey carton, atoms treated QM (Ser62, Lys65, Tyr153, Asn155, H_2_O and d-Ala-OEt) are shown in ball and stick representation (Asp481, also treated as QM, is not shown for clarity). The carbon atoms of the acyl-intermediate are shown in green color, carbon atoms of d-Ala-OEt are displayed in pale green. AI, acyl-intermediate; TS, transition state.

The aminolysis reactions for d-Ala-OEt and l-Ala-OEt follow the same process. First, the nucleophilic attack by the N atom of the d- or l-Ala-OEt to the C1 atom of the acyl intermediate occurs, reducing its distance from approximately 2 Å to ∼1.6 Å, resulting in the formation of a covalent bond (Fig. 6a, b, S7, S8a and b†). However, to form the TS and advance the reaction, one of the H atoms of the amino group of the Ala-OEt nucleophile must be near the oxygen of Tyr153. At the same time, the proton of Tyr153 must be close to Lys65 (Fig. 6c and S8b†). Notably, Asn155 interacts with Lys65, helping it accept an additional proton from Tyr153. Then, proton exchange occurs; Lys65 extract a proton from Tyr153, and immediately following this, Tyr153 receives a proton from the amino group of the Ala-OEt nucleophile (Fig. 6c, d, S8c and d†). The deprotonated nucleophile then binds to the acyl intermediate, forming the tetrahedral structure seen in the TS (Fig. 6e, f and S8e†). If the oxygen of Tyr153 is not close enough to the nucleophile to extract a proton, the TS would be unstable, the reaction would not take place and the N of the Ala-OEt will separate from the C1 atom of the acyl intermediate. After the formation of this tetrahedral species, protonated Lys65 moves closer to Ser62, and the covalent bond between the Ser62 oxygen and the C1 atom of the acyl intermediate stretches until it breaks (Fig. 6f, g, S8f and g†). Then, Ser62 accepted a proton from protonated Lys65 (Fig. 6g, h and S8†).

We speculate that Asn155 and the water molecule, which are close during the course of the reaction, help reduce the energy barrier of the proton transfer from the amino group of the nucleophile to Tyr153, stabilizing the tetrahedral intermediate dipeptide species that retains the two protons on its amino group. In addition, Asn155 seems to stabilize the charge on Lys65, allowing it to accept a proton from Tyr153. However, in the reactions with l- and d-Ala-OEt, the distance between the water molecule and the implicated residues (Asn155 and the amino group of the nucleophile) are not significantly different.

There are some differences between the two systems, one being the orientation of the side-chain amino group in the acyl intermediate (in both systems, the methyl group of the acyl intermediate is located in the hydrophobic pocket formed by the residues Ala226 and Trp220). In the reaction of d-Ala-OEt, the amino group of the acyl intermediate is located above the methyl group, allowing it to interact with Asp479 and the side chains of Ala430 and Ala482. On the other hand, the amino group of the acyl intermediate in the aminolysis reaction of l-Ala-OEt is at the same level as the methyl group. This allows the amino group to interact with Asp479 and form an additional interaction with Asn155, stabilizing this species. Another difference between these two reactions is the distance between His285 and Tyr153 in the active site. The methyl group of the l-Ala-OEt nucleophile is oriented towards His285, and due to steric hindrance, it does not allow His285 to be close to Tyr153 (Fig. S9†). On the other hand, in the system with d-Ala-OEt, the methyl group of the Ala-OEt nucleophile is not oriented in that direction, and His285 can be closer to Tyr153, forming a stabilizing interaction with Tyr153 (Fig. S9†). Another difference that can be observed between the reactions is the dihedral angle, psi (*ψ*), between the N atom of the nucleophile and the C1 atom in the formed dipeptide. The newly formed peptides displayed different phi (*φ*) and psi (*ψ*) dihedral angles due to the position of their methyl groups, which changed the angle of the nucleophile attack. d-Ala-OEt presents a *φ* dihedral angle of ∼−160 degrees, whereas l-Ala-OEt presents an angle of approximately −110 degrees (Fig. S10†). The *ψ* angle displayed by the l-alanine dipeptide was ∼−20 degrees, and the dihedral angle of the d-alanine dipeptide was ∼158 degrees. In the case of l-Ala-OEt, these dihedrals angles are within the allowed alpha region. In contrast, the d-alanine dipeptide dihedrals are not in the allowed regions for the majority of the reaction, as the methyl of the second alanine and the amino N-terminal group are *trans* to each other (Fig. 6h).^73^ This could partially explain the higher energy barrier for the conversion of the AI of the d-alanine dipeptide to the product (Fig. 5), but it does not account for the higher energy barrier of the aminolysis reaction involving l-Ala-OEt.

The energy obtained for the aminolysis reaction with DAP for d-Ala-OEt (28.8 kcal mol^−1^) is similar to that previously calculated for papain with the same substrate.^30^ However, in the case of l-Ala-OEt, the energy calculated here is significantly higher (44.5 kcal mol^−1^) than that found with papain (12 kcal mol^−1^).^30^ In fact, we note that the barrier of the papain-mediated aminolysis reaction for d-Ala-OEt was previously calculated to be 28 kcal mol^−1^, but papain cannot polymerize d-Ala-OEt. Here, we found that DAP is able to polymerize both substrates, but it is more efficient for d-Ala-OEt, which is consistent with the QM/MM results, but the energies presented here are higher than those presented for the papain-mediated polymerization.^30^ Recently, the hydrolysis of the desulfated inhibitor avivactam by a class-C β-lactamase was reported with an energy barrier of 40 kcal mol^−1^ and a *t*_1/2_ of 10^2^–10^4^ h.^71^ Based on these previous reports, we consider that the high-energy barriers we encountered in the aminolysis reaction, especially for l-Ala-OEt, could be overestimations. This overestimation could be due to several factors; the main reason is our inability to reliably replicate the reaction, as all the reactions modeled using different CVs failed or displayed higher energetic barriers. The other reasons are diverse but could include the selection of the QM atoms, which is critical for accurately modeling the reactions. The active site of DAP is buried inside the protein and forms an extensive network of contacts and H-bonds that are difficult to model at the QM level. As stated before, incorporating additional atoms into our QM initial systems reduced the energy barrier, as shown by our first attempts to model the aminolysis reaction with DAP. In general, as the QM region size increases, more accurate results can be obtained,^74^ but at the expense of increasing the computational calculation time. Thus, it is plausible that including more atoms in the QM region of our system would afford a more accurate model of the reaction. In addition, the selection of CVs to appropriately describe the reaction is known to be a critical step and a major limitation of metadynamics and similar techniques.^67^ Different reactions have been modeled using a variety of CVs with no positive results. However, despite our best effort, the selected CVs used to finally model the aminolysis reaction may not be accurate enough to fully describe this complex reaction.

Previous works by Asano *et al.* showed that DAP displays higher stereospecificity towards d-amino acid derivatives, amides and esters as well as peptides with a d-alanine at their N terminus than towards the corresponding l-amino acid derivatives.^31,32^ However, additional studies have shown that DAP can effectively polymerize d-alanine oligopeptides, although those reactions were performed in nonaqueous media.^38^ Using immobilized DAP in water-saturated toluene and in the presence of an organic tertiary amine, DAP was able to catalyze the polymerization of d-alanine oligomers to give the dimer and trimer in 56% and 6% yield, respectively, from d-alanine methyl ester as the monomer. Here, we used DAP in an aqueous buffer without an organic solvent or tertiary amine, showing its ability to polymerize amino acid monomers into short polypeptides under mild conditions. Despite the fact that DAP is able to polymerize l- and d-Ala-OEt, the experimental analysis confirmed that it clearly has a preference for dextrorotatory substrates. Although DAP can catalyze the formation of l-alanine dipeptide, its activity is ∼26 times lower than its activity with d-Ala-OEt as the substrate. Indeed, the free energy barriers obtained from the QM/MM simulations for the reactions are in accordance with the experimental evidence. The barriers for the acylation (37.2 kcal mol^−1^) and aminolysis (44.5 kcal mol^−1^) with l-Ala-OEt are, in both cases, higher than the corresponding barriers for d-Ala-OEt (Table 1). d-Ala-OEt seems to be able to form more interactions with residues in the active site of the enzyme, such as with the conserved residues Asn155 and Asp479, and these interactions do not form in the acylation with l-Ala-OEt, and the methyl group of d-Ala-OEt is located closer to a hydrophobic pocket. The water molecules present in the active site are also likely involved in the aminolysis reaction, stabilizing the acyl intermediate and facilitating proton transfer between residues. In the aminolysis, the acyl intermediate seems more stable in the reaction with l-Ala-OEt, as the amino group interacts with Asp479 and Asn155; this last interaction is absent in the case of the AI with the dextrorotatory isomer. Thus, DAP preferring d-substrates can be attributed to chirality.

## Conclusions

In this study, we demonstrated the DAP-mediated polymerization of d-Ala-OEt in an aqueous solution. The present results showed that DAP has higher activity with d-Ala-OEt than with l-Ala-OEt. The QM/MM ABMD results were in agreement with the experimental results. The calculated energy landscapes indicate that both the d- and l-substrates underwent concerted reactions, with aminolysis being the limiting step in the catalytic step because it has higher activation energies for both substrates. The reaction of l-Ala-OEt with DAP displayed higher energy barriers for both acylation and aminolysis relative to those of the d-Ala-OEt substrate. In the acylation reaction, the interaction between the amino group of the substrate with Asn155 reduced the energetic barrier faced by d-Ala-OEt, whereas in the aminolysis reaction, the orientation of the amino group in l-Ala-OEt towards Asp479 and Asn155 stabilized this species, and the orientation of the attacking nucleophile could increase the energetic barrier relative to that with d-Ala-OEt. The combined experimental and computational results could provide a platform for synthesizing polypeptides that incorporate d-amino acids and help clarify the stereospecificity of proteases. The possibility of using DAP in aqueous buffers without organic solvents broadens the applications chemoenzymatic synthesis and allows the incorporate d-amino acids as substrates. With their polymerization and incorporation into polypeptides, more biostable peptides could be achieved, and such species may offer benefits for biotechnological and pharmaceutical industries for use as inhibitors or delivery systems.

## Conflicts of interest

There are no conflicts to declare.

## Supplementary Material

RA-010-D0RA01138J-s001
