# Supplementary material for: Computational study on the polymerization reaction of d-aminopeptidase for the synthesis of d-peptides
Source: RSC Adv. 2020 May 6;10(30):17582–92. doi: 10.1039/d0ra01138j (PMC9053604; doi:10.1039/d0ra01138j)
Supplement: RA-010-D0RA01138J-s001 [file RA-010-D0RA01138J-s001.pdf]

Electronic Supplementary Material (ESI) for RSC Advances.
This journal is © The Royal Society of Chemistry 2020

ELECTRONIC SUPPLEMENTARY INFORMATION

D-Aminopeptidase for the synthesis of D-peptides: an experimental and computational study

Joan Gimenez-Dejoz,[a] Kousuke Tsuchiya,[a] Ayaka Tateishi,[a] Yoko Motoda,[a] Takanori Kigawa,[b] Yasuhisa Asano,[b] Keiji Numata*,[a]

[a]Biomacromolecules Research Team, RIKEN Center for Sustainable Resource Science, 2-1 Hirosawa, Wako-shi, Saitama 351-0198, Japan.
[b]Laboratory for Cellular Structural Biology, RIKEN Center for Biosystems Dynamics Research, 1 -7-22 Suehirocho, Tsurum, Yokohama, Japan.
[c]Biotechnology Research Center, Department of Biotechnology, Toyama Prefectural University, 5180 Kurokawa, Imizu, Toyama 939-0398, Japan.

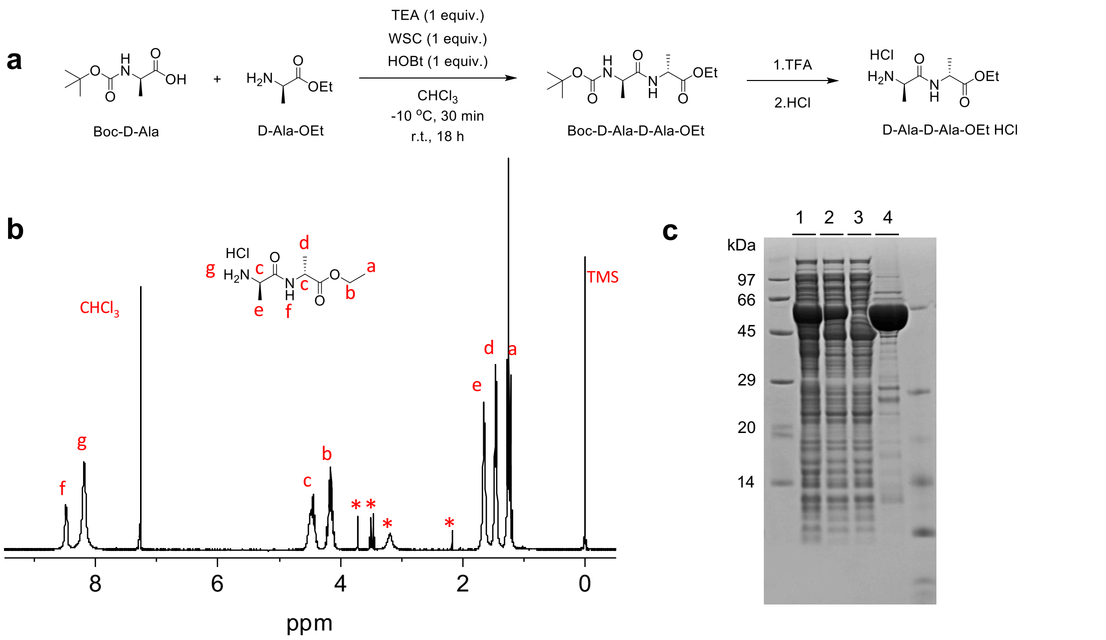

Figure S1. Reaction scheme of the synthesis of D-Alanine dipeptide ethyl ester (a). ^{1}H NMR spectra of D-Alanine dipeptide as standard (b). Peaks marked with an asterisk are peaks corresponding to solvent. (c) SDS-gel analysis of the purification process. Lane 1; total fraction, 2; supernatant fraction, 3; column flow through, 4; Histrap elution fraction.

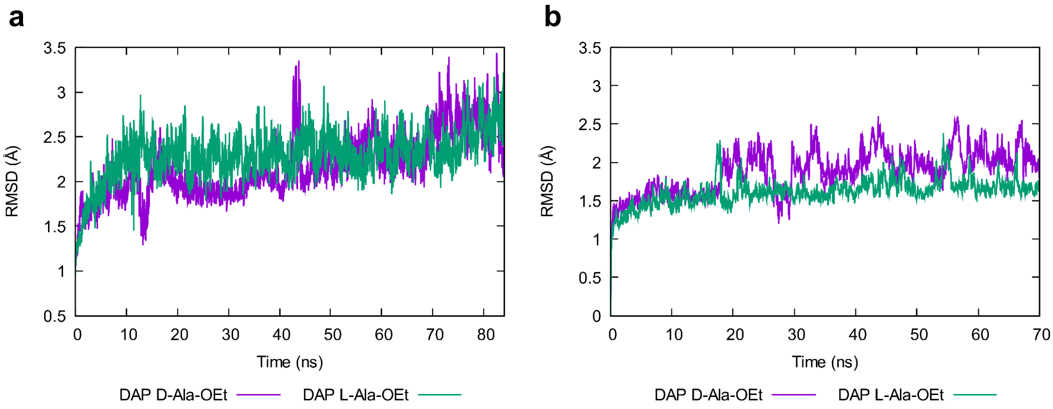

Figure S2. RMSD of DAP equilibrated with classical MD for modelling the acylation (a) and aminolysis (b) reactions.

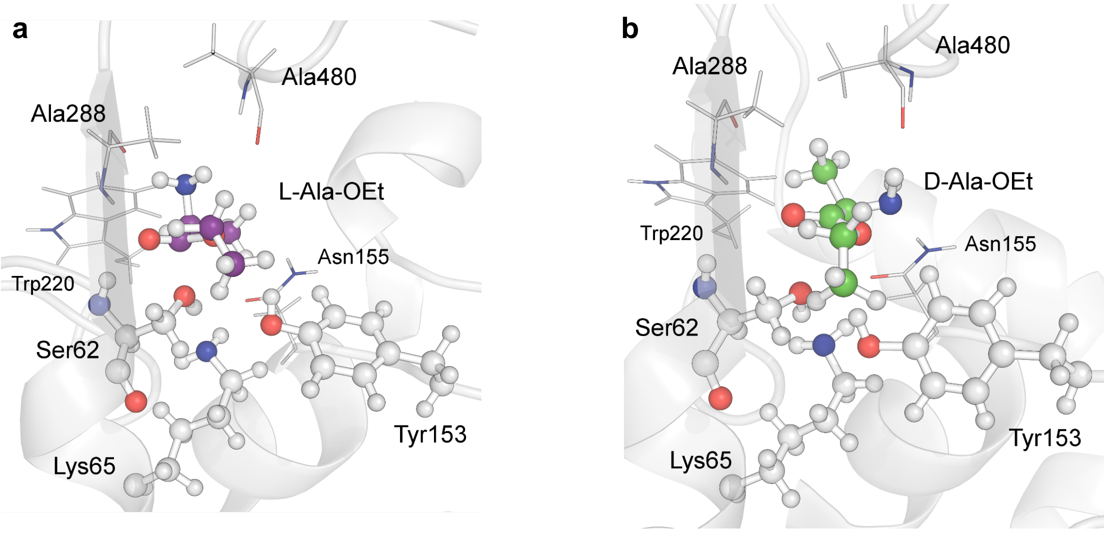

Figure S3. Starting configuration of the acylation reaction for L-Ala-OEt (a) and D-Ala-OEt (b) showing all the atoms included in the QM region. DAP is represented in grey cartoon. Atoms included in the QM region are represented in ball and stick representation. Important residues nearby the substrate are represented as sticks. Water molecules and parts of DAP are not shown for clarity.

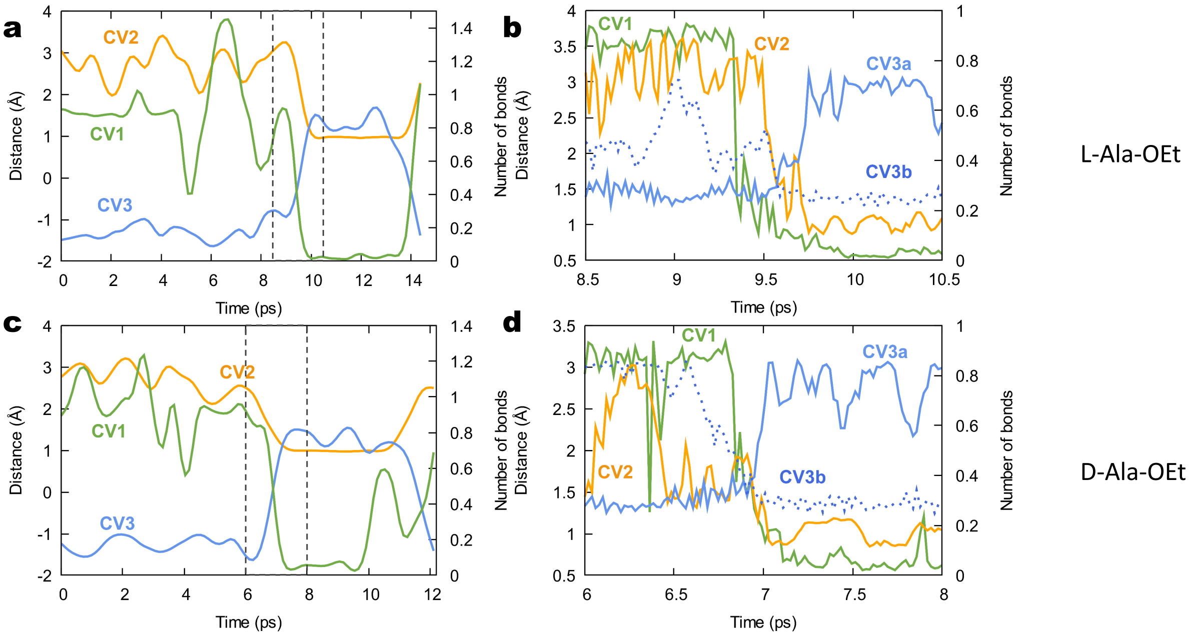

Figure S4. Time evolution of the CVs along the all the acylation reaction time (A,C) and enlarged view of the CV around the TS (b,d), for the acylation reaction with L-Ala-OEt (top) and with D-Ala-OEt (bottom). CV1, as number of bonds, is displayed as a green line. CV2 and CV3 distances are displayed as orange and blue lines, respectively. In B and D, CV3 is divided in the original combination of distances, CV3a (blue line) and CV3b (doted blue line). CVs are color coded according to text and Figure 1a.

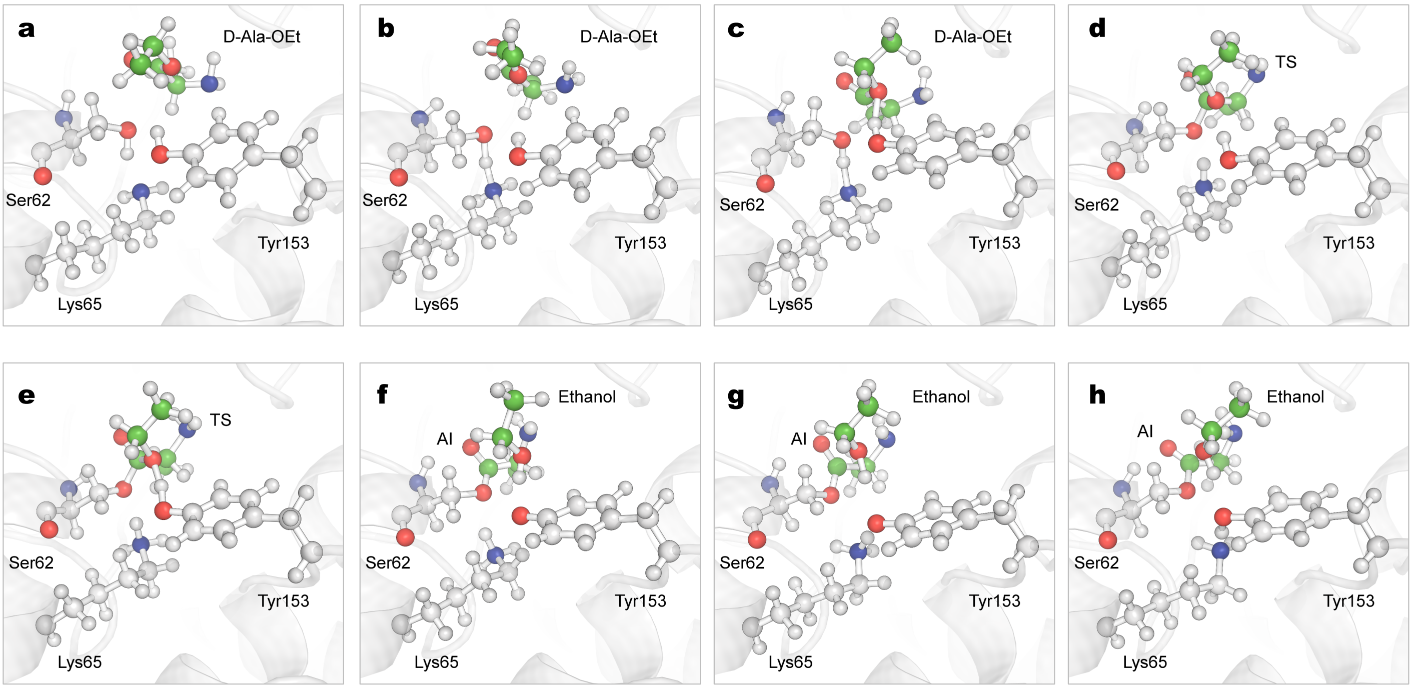

Figure S5. Mechanism of the acylation reaction of DAP and D-Ala-OEt obtained from QM/MM ABMD simulations. DAP is represented in grey cartoon, atoms treated QM (Ser62, Lys65, Tyr153 and L-Ala-OEt) are shown in ball and stick representation. The carbon atoms of the D-Ala-OEt are shown in green color. AI, acyl-intermediate; TS, transition state.

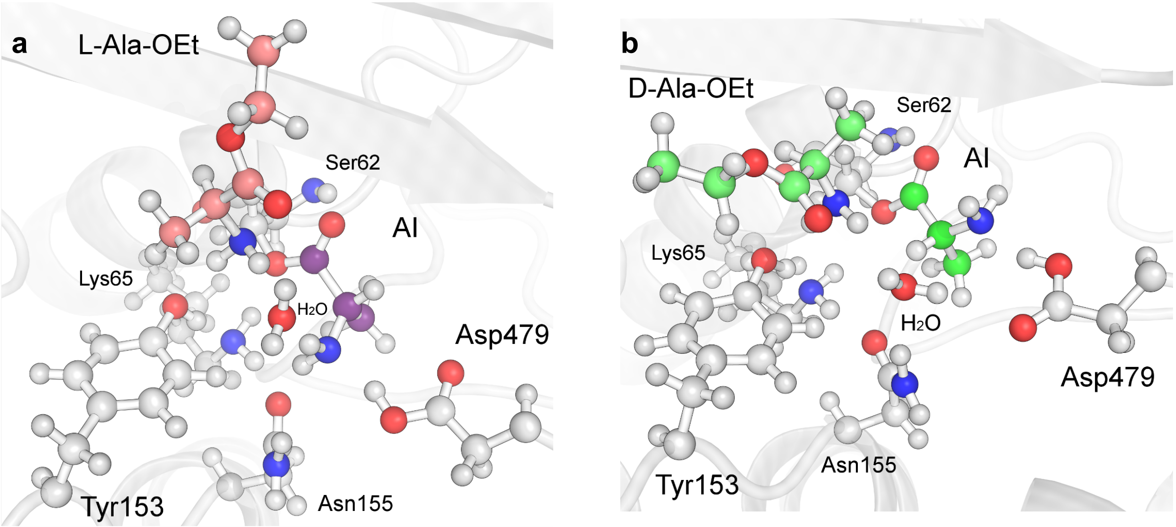

Figure S6. Starting configuration of the aminolysis reaction for L-Ala-OEt (a) and D-Ala-OEt (b) showing all the atoms included in the QM region. Atoms included in the QM region are represented in ball and stick representation, atoms of DAP are in grey, acyl-intermediate (AI) are in purple and green for L- and D- reactions, respectively. Atoms of the attacking nucleophile are displayed in salmond for L-Ala-OEt and pale green for D-Ala-OEt. DAP is represented in grey cartoon. Water molecules are not shown for clarity. AI, acyl-intermediate.

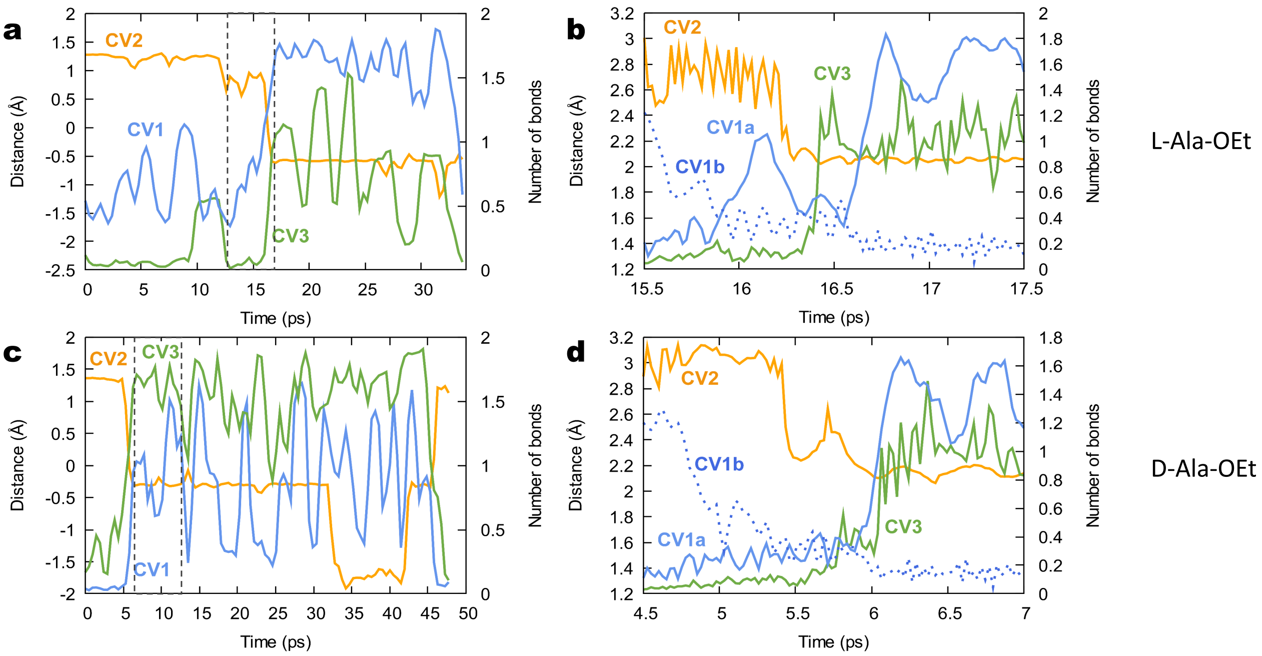

Figure S7. Time evolution of the CVs along the all the aminolysis reaction time (A,C) and enlarged view of the CV around the TS (B,D), with L-Ala-OEt (top) and D-Ala-OEt (bottom). CV2 and CV3, as number of bonds, are displayed as an orange and green line, respectively. CV1 as a LCOD is displayed in blue lines. In B and D, CV1 is divided in the original combination of distances, CV1a (blue line) and CV1b (doted blue line). CVs are color coded according to text and Figure 1b.

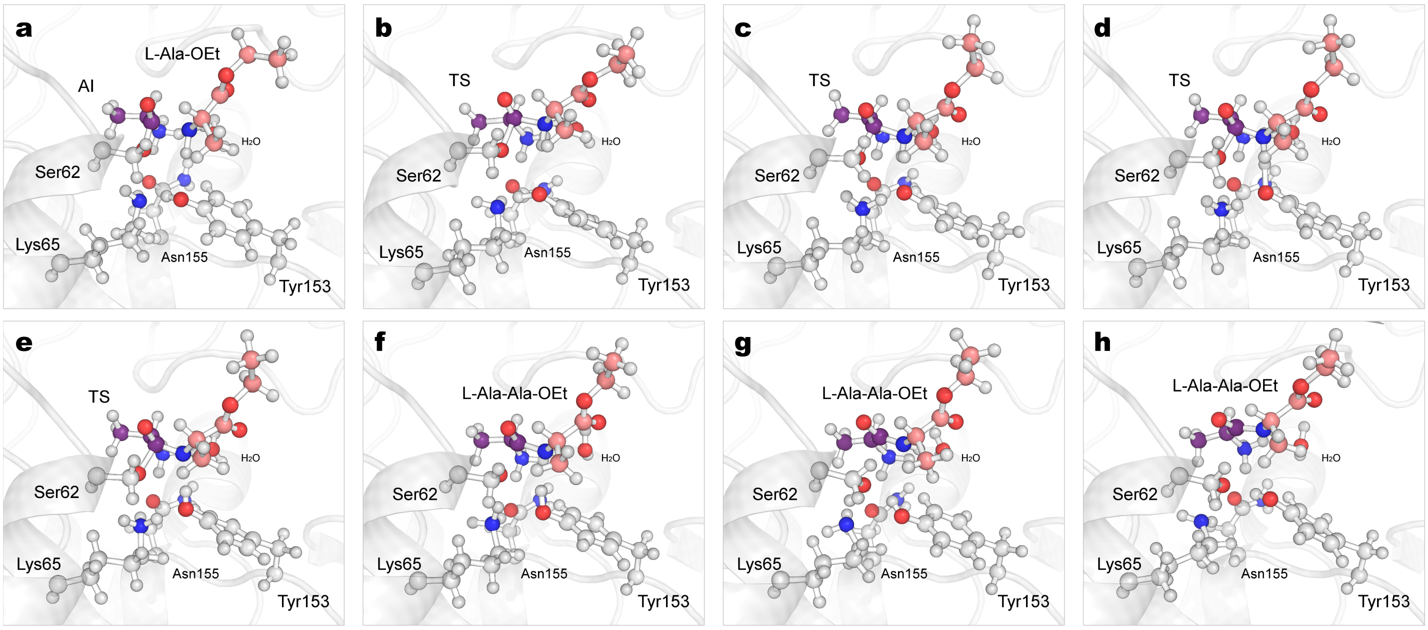

Figure S8. Mechanism of the aminolysis reaction of DAP with Ser62-acylated and L-Ala-OEt obtained from QM/MM ABMD simulations. DAP is represented in grey carton, atoms treated QM (Ser62, Lys65, Tyr153, Asn155, H2O and L-Ala-OEt) are shown in ball and stick representation (Asp481, also treated as QM, is not shown for clarity). The carbon atoms of the acyl-intermediate are shown in dark-purple color, carbon atoms of L-Ala-OEt are displayed in salmon color. AI, acyl-intermediate; TS, transition state.

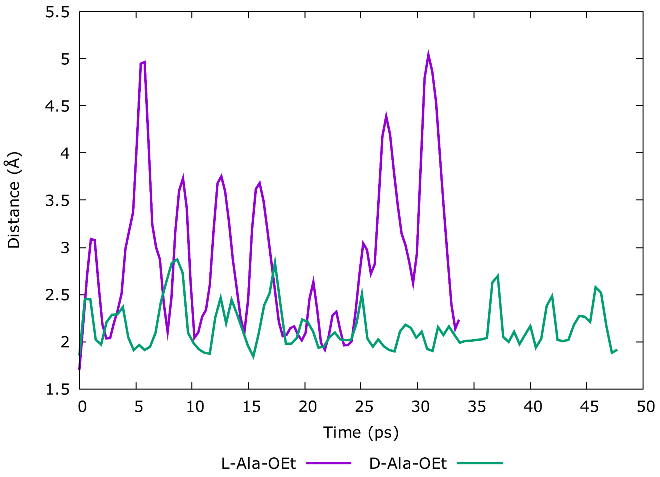

Figure S9. Distance between the Tyr153 oxygen and His285 Hε atom along the reaction time.

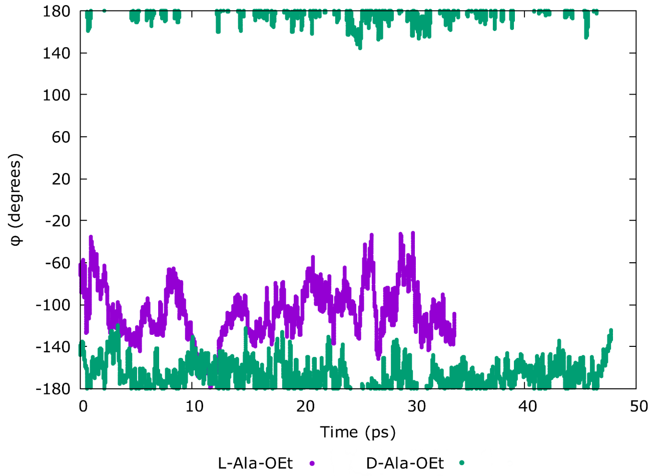

Figure S10. Dihedral ϕ angle between the attacking nucleophile and the AI and of the newly formed dipeptides during the reaction time. AI, acyl-intermediate.
